# The ACVR1 R206H mutation found in fibrodysplasia ossificans progressiva increases human induced pluripotent stem cell-derived endothelial cell formation and collagen production through BMP-mediated SMAD1/5/8 signaling

**DOI:** 10.1186/s13287-016-0372-6

**Published:** 2016-08-17

**Authors:** Emilie Barruet, Blanca M. Morales, Wint Lwin, Mark P. White, Christina V. Theodoris, Hannah Kim, Ashley Urrutia, Sarah Anne Wong, Deepak Srivastava, Edward C. Hsiao

**Affiliations:** 1 Institute for Human Genetics and the Division of Endocrinology and Metabolism, University of California, 513 Parnassus Avenue, HSE901G, San Francisco, CA 94143-0794 USA; 2 Gladstone Institute of Cardiovascular Disease, 1650 Owens Street, San Francisco, CA 94158 USA; 3 School of Dentistry, Oral and Craniofacial Sciences Program, University of California, 707 Parnassus Avenue, San Francisco, CA 94143 USA; 4 Department of Endocrinology, Diabetes, and Metabolism, Institute for Human Genetics, University of California, 513 Parnassus Avenue, HSE901G, UCSF Box 0794, San Francisco, CA 94143-0794 USA

**Keywords:** ACVR1, Fibrodysplasia ossificans progressiva, Tissue fibrosis, hiPS-derived endothelial cells, BMP, Activin A signaling

## Abstract

**Background:**

The Activin A and bone morphogenetic protein (BMP) pathways are critical regulators of the immune system and of bone formation. Inappropriate activation of these pathways, as in conditions of congenital heterotopic ossification, are thought to activate an osteogenic program in endothelial cells. However, if and how this occurs in human endothelial cells remains unclear.

**Methods:**

We used a new directed differentiation protocol to create human induced pluripotent stem cell (hiPSC)-derived endothelial cells (iECs) from patients with fibrodysplasia ossificans progressiva (FOP), a congenital disease of heterotopic ossification caused by an activating R206H mutation in the Activin A type I receptor (ACVR1). This strategy allowed the direct assay of the cell-autonomous effects of ACVR1 R206H in the endogenous locus without the use of transgenic expression. These cells were challenged with BMP or Activin A ligand, and tested for their ability to activate osteogenesis, extracellular matrix production, and differential downstream signaling in the BMP/Activin A pathways.

**Results:**

We found that FOP iECs could form in conditions with low or absent BMP4. These conditions are not normally permissive in control cells. FOP iECs cultured in mineralization media showed increased alkaline phosphatase staining, suggesting formation of immature osteoblasts, but failed to show mature osteoblastic features. However, FOP iECs expressed more fibroblastic genes and Collagen 1/2 compared to control iECs, suggesting a mechanism for the tissue fibrosis seen in early heterotopic lesions. Finally, FOP iECs showed increased SMAD1/5/8 signaling upon BMP4 stimulation. Contrary to FOP hiPSCs, FOP iECs did not show a significant increase in SMAD1/5/8 phosphorylation upon Activin A stimulation, suggesting that the ACVR1 R206H mutation has a cell type-specific effect. In addition, we found that the expression of *ACVR1* and type II receptors were different in hiPSCs and iECs, which could explain the cell type-specific SMAD signaling.

**Conclusions:**

Our results suggest that the ACVR1 R206H mutation may not directly increase the formation of mature chondrogenic or osteogenic cells by FOP iECs. Our results also show that BMP can induce endothelial cell dysfunction, increase expression of fibrogenic matrix proteins, and cause differential downstream signaling of the ACVR1 R206H mutation. This iPSC model provides new insight into how human endothelial cells may contribute to the pathogenesis of heterotopic ossification.

**Electronic supplementary material:**

The online version of this article (doi:10.1186/s13287-016-0372-6) contains supplementary material, which is available to authorized users.

## Background

Diseases of heterotopic ossification, where bone forms at an abnormal site, provide valuable opportunities to examine the mechanisms that regulate osteogenesis. The bone growth can range from small incidental nodules to catastrophic paralysis. Heterotopic ossification is found in a wide variety of diseases, including traumatic injury, severe burns, brain injury, and invasive surgeries [1–3]. However, the diversity of triggers poses a significant challenge for dissecting the cellular and molecular mechanisms that cause heterotopic ossification. Thus, genetic conditions of abnormal bone formation provide a valuable model for identifying the key pathways and regulators of heterotopic ossification in soft tissues.

Fibrodysplasia ossificans progressiva (FOP) is a congenital disease characterized by a large amount of heterotopic ossification in postnatal muscles and tendons [4, 5] and has been used as a prototypical model for studying heterotopic ossification. Most patients have a conserved mutation in the Activin A type 1 (*ACVR1*/*ALK2*) gene [6–8], a bone morphogenetic protein (BMP) receptor. BMPs are major regulators of bone formation. They were initially identified based on their ability to induce bone formation in soft tissues such as muscle and tendon. The majority of ACVR1 mutations in FOP are localized to a single amino acid change (R206H) that is thought to increase ACVR1 signaling activity.

Histologically, the initial fibrocellular infiltrate at a bone formation site is fibrotic and contains inflammatory cells and endothelial cells (ECs) [7]. ECs overexpressing ACVR1 R206H may contribute to heterotopic ossification [9], possibly via abnormal BMP signaling, by undergoing endothelial-to-mesenchymal transition (EndMT) [10]. However, it is unclear if human FOP ECs can transdifferentiate into osteogenic cells and directly contribute to bone formation, if another mechanism leads to the increase in ECs in FOP lesions [7, 9], if ECs are crucial in the very early steps of heterotopic ossification, or if human FOP ECs also respond abnormally to BMP or Activin A signals acting on the ACVR1 R206H receptor [11, 12].

Studies on human skeletal development are hampered by a number of factors, including the rare access to fetal material of developing bones, the difficulty of obtaining primary cells for detailed laboratory analysis, the ubiquitous nature of many of the ligands, and the technical and ethical problems surrounding the potential human genetic experiments needed to test pathways and hypotheses. Given that primary tissues cannot be obtained from patients with FOP because of heterotopic ossification at the surgical site, patient-derived human induced pluripotent stem cells (hiPSCs) [13] provide a strategy to create FOP cells for study in vitro [14].

The recent development of hiPSCs has made genetic diseases easier to study. hiPSCs can be derived directly from patients with existing genetic mutations and can form any cell in the body. hiPSCs also allow us to study the effect of single mutations in the endogenous locus and in a cell-autonomous fashion without the inherent complications of transgenic or overexpression studies. Thus, iPSC-derived tissues can model early developmental events and provide diseased human tissues that cannot be obtained from a patient.

Here, we use hiPSCs from patients with FOP [14] as a model of cell-autonomous signaling induced by the ACVR1 R206H mutation to test if the mutation increases the formation of potential osteoprogenitors in the EC lineage, and if hiPSC-derived endothelial cells (iECs) expressing ACVR1 R206H show increased plasticity towards osteogenesis. We also investigate the cellular mechanisms by which ECs may contribute to heterotopic ossification.

## Methods

### Cell culture and differentiation

Pluripotent hiPSC lines derived from control (wild type, WT) and FOP fibroblasts previously described [14, 15] were maintained in mTeSR1 medium (StemCell Technologies) on irradiated SNL (mouse fibroblast STO cell line transformed with neomycin resistance and murine LIF genes) feeder cells [16]. SNLs were removed by at least one passage in feeder-free conditions on growth-factor-reduced Matrigel (Corning)-coated plates (150–300 μg/ml, 40 min coating) before use in differentiation assays. ROCK inhibitor Y-27632 (10 μM, StemCell Technologies) dissolved in 100 % DMSO was added to mTeSR1 at the time of passaging and removed the following day.

hiPSC lines were differentiated into iECs according to protocols previously described [17]. Embryoid bodies (EBs) were formed from hiPSCs and cultured in aggregation medium Stem-Pro-34 (Invitrogen) supplemented with 2 mM glutamine, 150 mg/ml transferrin, 1 mM ascorbic acid, and 0.4 μM monothioglycerol (Sigma-Aldrich). EBs were cultured in aggregation medium with 5 ng/ml human basic fibroblast growth factor (bFGF), 3.6 ng/ml Activin A, and 12 ng/ml BMP4 (Peprotech). On day 6 of differentiation, cells were sorted for endothelial progenitor markers PECAM and KDR and plated on fibronectin (Sigma-Aldrich) in endothelial cell medium (ECM, ScienCell). These iECs were able to be passaged up to three times. iEC cultures were also treated with the Activin A inhibitor follistatin at 1 μg/ml (Peprotech).

### Mineralization assay

hiPSCs maintained in feeder-free conditions were plated in 20 % mTeSR1 mixed with 80 % OB (osteoblastic mineralization) medium [Dulbecco’s Modified Eagle’s Medium with 20 % fetal bovine serum, 2 mM GlutaMAX^TM^, 10 mM glycerol-2-phosphate, 1 nM dexamethasone, 0.1 mM 2-mercaptoethanol, and 50 μg/ml L-ascorbic acid 2-phosphate sesquimagnesium salt hydrate, 1 % non essential amino acids] and Y-27632 (10μM) at 400,000 cells per well in gelatin-coated 24-well plates [14]. The medium was replaced on day 2 with 100 % OB medium. The medium was changed every other day until day 12. Samples for the alkaline phosphatase staining were fixed on day 15 in 95 % ethanol for 10 min to 1 h at room temperature and incubated with substrate BCIP/NBT (Sigma-Aldrich) for 10 min at 37 °C. Staining was quantified via Image J [18].

### Reverse Transcriptase-PCR and quantitative expression analysis

Total RNA was prepared using TRI Reagent (Sigma-Aldrich) and treated with the Turbo DNA-Free kit (Ambion). One microgram of total RNA was reverse transcribed into cDNA with the iScript cDNA Synthesis kit (BioRad) according to manufacturer instructions. Real-time quantitative PCR was performed in triplicate with VeriQuest Probe qPCR Master Mix (Affymetrix) or ABI’s Sybr Green PCR Master Mix on a ViiA 7 thermocycler (Life Technologies). Some low-yield cDNA samples were pre-amplified and analyzed using the BioMark 48.48 Dynamic Array nanofluidic chip (Fluidigm Inc., USA) according to manufacturers’ instructions. Taqman primer and Sybr Green probe sets are listed in Additional file 1: Table S1 and Additional file 2: Table S2. *GAPDH* or *β-actin* was used for normalization as an endogenous control.

### Immunostaining

iECs were fixed with 4 % paraformaldehyde/phosphate-buffered saline for 10 min at room temperature, then blocked with 5 % bovine serum albumin. Cells were stained overnight with primary antibodies to PECAM (5 μg/ml, R&D Systems) and VE-Cadherin (2 μg/ml, R&D Systems). Secondary antibodies were from Life Technologies: Alexa488-conjugated goat anti-mouse IgG (1:500) and Alexa555-conjugated goat anti-rabbit IgG (1:500). Nuclei were stained with DAPI in the ProLong® Gold Antifade (Life Technologies) mounting media. Images were taken using a light microscope (Nikon Eclipse E800 or Leica DMI 4000B).

### Flow cytometry

hiPSCs cultured in mineralization medium were dissociated into single cells with collagenase type I (Worthington) for 1 h at 37 °C and then isolated by a Ficoll gradient (Histopaque 1191, Sigma-Aldrich). Accutase was used to generate single-cell suspensions from EBs plated overnight on collagen IV-coated plates or from iECs grown on fibronectin-coated plates. Cells were stained with PECAM1-AF488, KDR-APC, and VE-Cadherin-PerCP-Cy5.5 antibodies for endothelial markers, with CD90-AF488, CD73-PE, and CD105-PerCP-Cy5.5 antibodies (all from BD Pharmingen) for mesenchymal stem cell (MSC) markers. ICAM-1-PE antibody was used in our TNFα induction assay. Fluorescence intensity was determined for 10,000 cells in total and percentages shown in figures are the percentage of living cells that fall within the gate shown.

### Vascular tube formation assay

Cells were seeded at 2.5 × 10^5^ per well on 24-well plates pre-coated with growth factor-reduced Matrigel (Corning) and incubated for 24 h at 37 °C. Images were taken using a light microscope (Nikon and Leica).

### ELISA

Activin A levels were measured in iEC culture supernatant using an immunoassay solid-phase ELISA (R&D Systems). Samples were assayed in biological triplicates.

### Western blot

iECs were plated on fibronectin following sorting at a density of 7.5 × 10^4^ cells per well of a 6-well plate and grown for 3 days in ECM medium (ScienCell). iECs were serum-starved for 1 h before a 40 min treatment with either 50 ng/ml of BMP4 or Activin A (R&D Systems). Cells were harvested in RIPA buffer (Pierce, Thermo Scientific) supplemented with 1X protease and a phosphatase inhibitor cocktail (Roche). Whole-cell lysates were prepared in Laemmli buffer (BioRad) and resolved in 4–20 % tris-glycine gels (BioRad). Primary antibodies towards SMAD1/5/8 (Santa Cruz Biotechnology), phospho-SMAD1/5/8 (Cell Signaling), SMAD2/3 (Cell Signaling), and phospho-SMAD2/3 (Cell Signaling) were used at a dilution of 1:1000. Anti-GAPDH antibody (Thermo Scientific) was used at a dilution of 1:10,000. Binding was visualized with horseradish peroxidase-conjugated antibodies (Cell Signaling) and ECL (Enhanced ChemiLuminescence) substrate (Thermo Scientific). An ImageQuant LAS 4000 (GE Healthcare) was used to image the blots and quantifications were done using Image J software.

### TNFα activation assay

Cells were seeded at 1 × 10^5^ per well on 6-well plates and treated with 10 ng/ml TNFα overnight at 37 °C in 5 % CO_2_. Cells were then harvested and stained for ICAM-1 and PECAM for fluorescence-activated cell sorting (FACS) analysis as discussed below.

### Transwell assay

For the transwell assay, 1 × 10^5^ cells were added onto each transwell inserts (8.0 μm pore, Sigma-Aldrich). The inserts were incubated for 24 h at 37 °C in 5 % CO_2_ with serum-free media, with or without 50 ng/ml VEGF in the lower chamber. Membranes from each insert were fixed for 20 min and stained with crystal violet for 1 h.

### Statistical analysis

Because each hiPSC line was derived clonally, and thus may display different behaviors, we treated each cell line as an individual biological replicate and pooled our results into control or FOP categories. All statistical analyses were completed using GraphPad Prism software. *P* values were calculated using the Student’s *t* test. *p* values ≤ 0.05 were considered statistically significant and are shown in the figures. Non-significant *p* values are not indicated for figure clarity.

## Results

### WT and FOP hiPSCs can form endothelial cells with equal efficiency in a directed differentiation protocol

Multiple signaling molecules, including BMPs [10], regulate EC formation. Also, human umbilical vein endothelial cells (HUVECs) overexpressing *ACVR1* R206H can acquire MSC-like phenotypes via EndMT [9]. We previously found that FOP hiPSCs show increased mineralization compared to control hiPSCs when cultured in mineralizing conditions [14]. To determine if the *ACVR1* R206H mutation could increase the formation of ECs during mineralization, we examined the expression of two EC markers, *PECAM* and *VE-Cadherin* (markers of mature ECs), at two time points during culture (Additional file 3: Figure S1A). *PECAM* and *VE-Cadherin* expression showed only a trend towards increased expression in the FOP cultures. Since this could occur from a dilution effect from non-ECs, we used FACS to quantitate cells with PECAM and KDR, which marks endothelial progenitors [17], and cells co-expressing MSC markers CD90, CD73, and CD105. We found increased PECAM^+^/KDR^+^ cells (ECs, Additional file 3: Figure S1B) but not increased CD90^+^/CDE73^+^/CD105^+^ cells (MSCs, Additional file 3: Figure S1C), suggesting that FOP hiPSCs cultured in mineralizing conditions increased EC lineages but not MSC lineages [14].

**Fig. 1:**
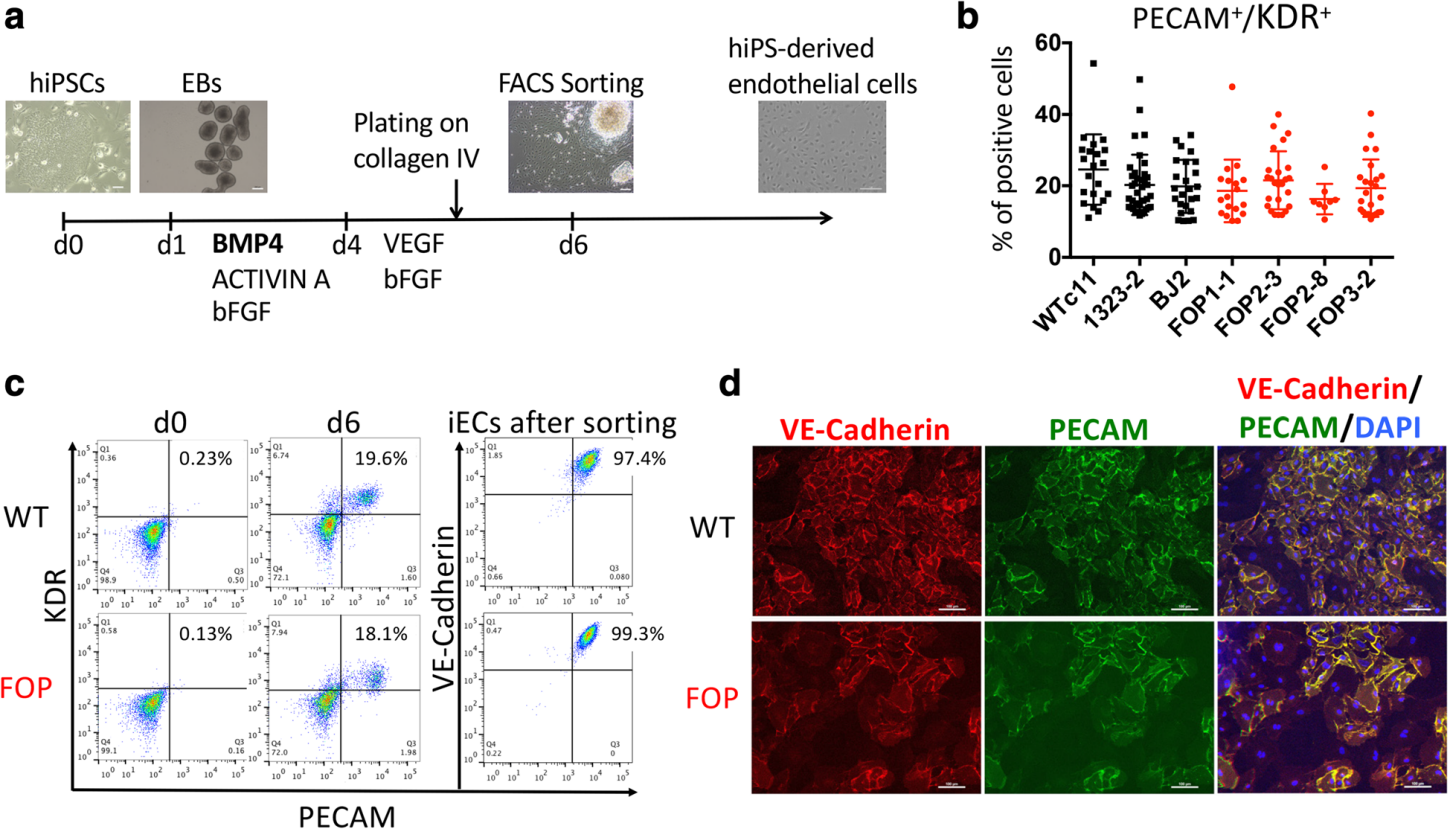
Differentiation of human induced pluripotent stem cell (hiPSC)-derived endothelial cells (iECs) from wild type (WT) and fibrodysplasia ossificans progressiva (FOP) hiPSC lines. **a** Endothelial cell differentiation protocol adapted from White et al. [17]. WT and FOP hiPSCs were induced to form mesodermal progenitors using BMP4, Activin A, and bFGF. Embryoid bodies (EBs) were cultured in medium supplemented with VEGF and bFGF on day 4 and plated onto collagen IV on day 5. iEC precursors were identified by KDR/PECAM positivity. Scale bars, 200 μm (hiPSCs, EBs, and EBs at day 6) and 1 μm (hiPS-derived endothelial cells). **b** Mean percentage of cells expressing both PECAM and KDR by fluorescence-activated cell sorting (FACS) analysis on day 6 of endothelial differentiation of one WT hESC line, three WT hiPSC lines, and four FOP hiPSC lines. Error bars represent the mean ± one standard deviation of at least three independent replicates for each of the three WT and four FOP cell lines. Mean values were not statistically different. **c** FACS analysis of WT and FOP hiPSCs on day 0 and day 6 of endothelial differentiation. iECs co-expressing PECAM and KDR were sorted by FACS on day 6 and then cultured in endothelial cell medium. Analysis of PECAM and VE-Cadherin expression by FACS of sorted iECs after one passage is shown on the *right*. **d** WT and FOP hiPSCs were differentiated as in Fig. 1A and sorted on day 6. iECs were immunostained for endothelial markers PECAM and VE-Cadherin. Scale bars, 100 μm

We next created iECs to test if FOP heterotopic ossification could arise from increased formation of skeletal precursors in the EC lineage without overexpression of *ACVR1* R206H (Fig. 1a) [17]. These conditions, which contain BMP4, produced a consistent yield of approximately 20 % phenotypic iECs (PECAM^+^/KDR^+^) from both WT and FOP hiPSC lines (Fig. 1b). These findings suggest that the R206H mutation does not impair or favor iEC progenitor formation from hiPSCs in our protocol.

### WT and FOP iECs show no functional differences

**Fig. 2:**
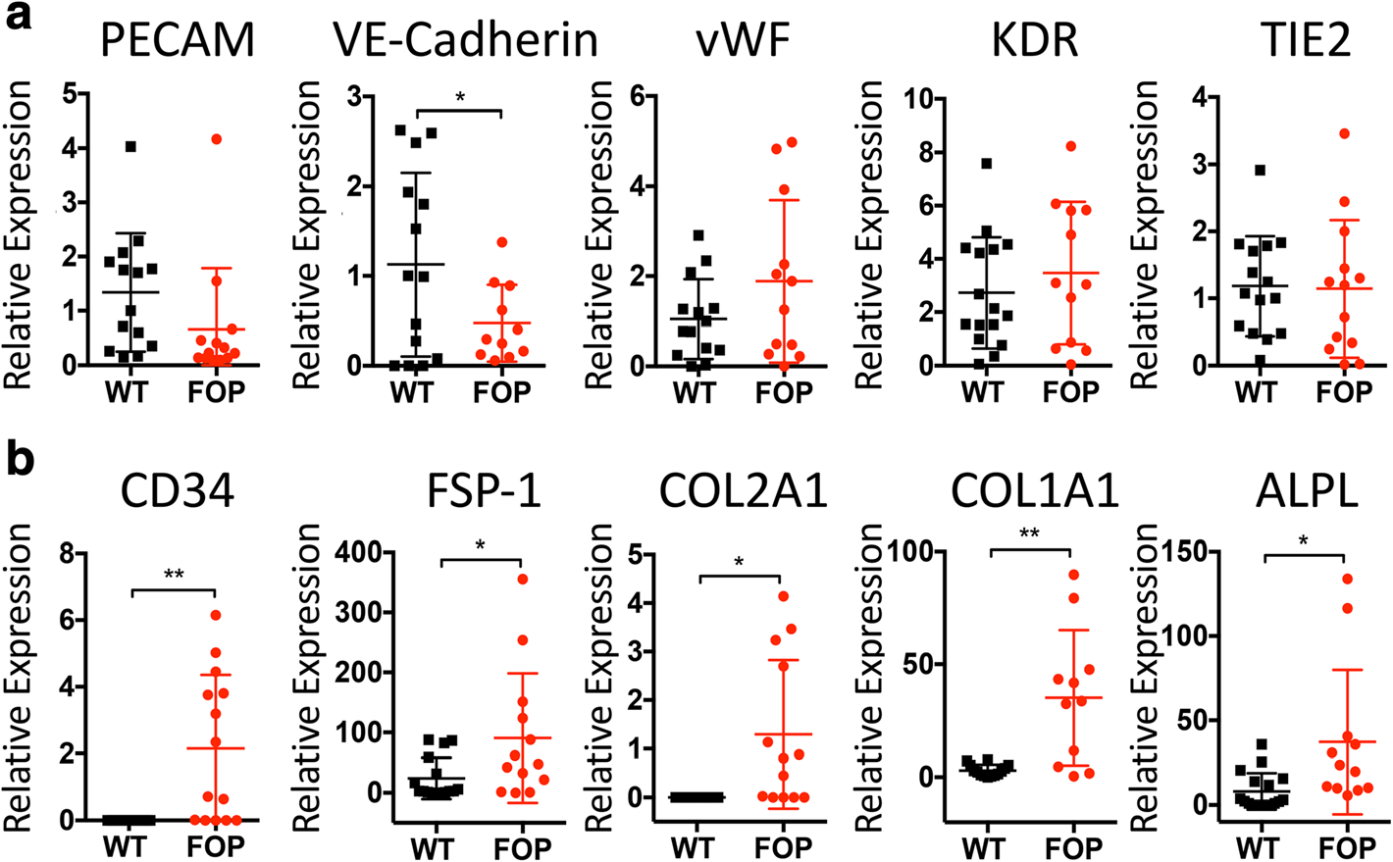
Loss of endothelial lineage commitment of fibrodysplasia ossificans progressiva (FOP) human induced pluripotent stem cell-derived endothelial cells (iECs). **a** Gene expression analysis of endothelial markers by quantitative PCR of wild type (WT) and FOP iECs. **b** Mesenchymal/fibroblastic (*FSP-1*, *CD34*), mature chondrogenic (*COL2A1*), and osteogenic (*COL1A1*, *ALPL*) gene expression were statistically different by Student’s *t* test: * *p* < 0.05, ** *p* < 0.01. Error bars represent the mean ± one standard deviation of at least three independent replicates for each of the three WT and four FOP iEC lines

To characterize our FOP iECs, we next purified the iECs and cultured them in vitro to assess their endothelial properties (Fig. 1a, c, d). After sorting, hiPSC-derived PECAM^+^/KDR^+^ iECs yielded more than 95 % PECAM^+^/VE-Cadherin^+^ proliferative cells (Fig. 1c), could be passaged up to three times (data not shown), and retained their endothelial phenotype (Fig. 1d). WT and FOP iECs formed vessel-like structures when cultured on Matrigel (Additional file 4: Figure S2A), showed increased ICAM-1 upon TNFα activation (Additional file 4: Figure S2B), and had similar migration properties to VEGF in transwells (Additional file 5: Figure S3). Cells showed equivalent mRNA levels of the endothelial markers *PECAM*, *vWF*, *KDR*, and *TIE2* (Fig. 2a). VE-Cadherin expression was significantly lower in FOP iECs, suggesting a partial loss of mature endothelial marker expression. Conversely, *CD34* mRNA, a marker for hematopoietic stem cell progenitors or vascular endothelial progenitors and also expressed on MSCs [19], was significantly increased in FOP iECs (Fig. 2b). These results indicate no significant differences in functionality between WT and FOP iECs but that FOP iECs show increased expression of an endothelial progenitor-like marker and decreased expression of one mature endothelial gene.

### FOP iECs show increased expression of alkaline phosphatase and Collagen 1/2

Since overexpression of R206H *ACVR1* can activate EndMT to create MSC-like cells from HUVECs [9, 20], and vascular-associated cells such as pericytes can form osteoblasts [21], we tested if FOP iECs could be directed towards osteogenesis. Chondrogenic and osteogenic gene expression profiling of purified iECs cultured in ECM showed elevated *COL1A1* and *COL2A1* (Fig. 2b), two gees that code for extracellular matrix proteins found in bone and cartilage. FOP iECs also expressed higher levels of the mesenchymal marker *FSP-1* gene (Fig. 2b), consistent with a predisposition towards EndMT. Some cultures of iECs showed increased levels of alkaline phosphatase (*ALPL*) mRNA (Fig. 2b), which was unexpected as the iECs were maintained in endothelial culture conditions. Although *ALPL* can mark immature osteoblasts, expression of chondrogenic and osteogenic transcription factors ( *SOX9*, *SP7*, and *RUNX2*) were unchanged in FOP iECs cultured in ECM (Additional file 6: Figure S4).

**Fig. 3:**
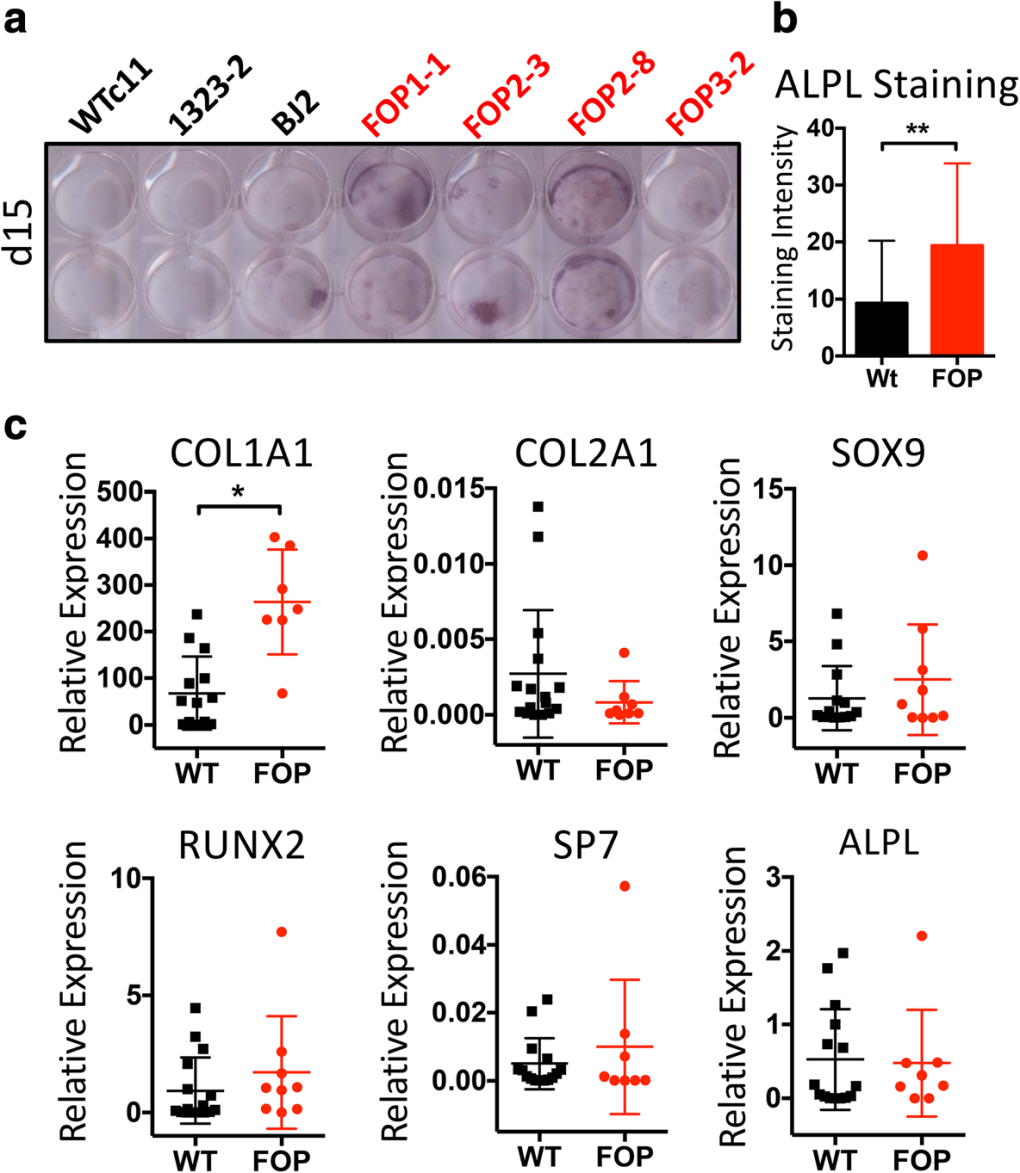
Osteogenic capacity of fibrodysplasia ossificans progressiva (FOP) human induced pluripotent stem cell-derived endothelial cells (iECs). **a** FOP iECs showed increased alkaline phosphatase (ALPL) staining at day 15. **b** Quantification of ALPL staining from at least three independent replicates for each of the three wild type (WT) and four FOP iECs lines. Error bars represent the mean ± one standard deviation; ** *p* < 0.01 by Student’s *t* test. **c** Osteogenic and chondrogenic gene expression analysis by quantitative PCR of WT and FOP iECs cultured in 50 % endothelial cell medium/50 % OB medium for 15 days. *COL1A1* and *COL2A1* gene expression were statistically different by Student’s *t* test: * *p* < 0.05. Error bars represent the mean ± one standard deviation of at least three independent replicates for each of the three WT and four FOP iEC lines

We next cultured our iECs in mineralization conditions to test if FOP iECs could form mineral-depositing cells via EndMT. After 15 days of culture, ALPL staining was increased in FOP iECs (Fig. 3a, b), again suggesting presence of osteogenic precursors; however, neither WT nor FOP iECs showed significant mineral deposition by von Kossa staining (data not shown), suggesting that mineralizing osteoblastic cells were not present. Gene expression analysis showed no difference in osteogenic markers except for increased *COL1A1* expression in FOP iECs (Fig. 3c). These results suggest that the ACVR1 R206H mutation may not directly increase osteogenic or chondrogenic potential in FOP iECs, but may increase extracellular matrix production and local tissue fibrosis.

### FOP iECs show increased formation in conditions not permissive for WT cells

**Fig. 4:**
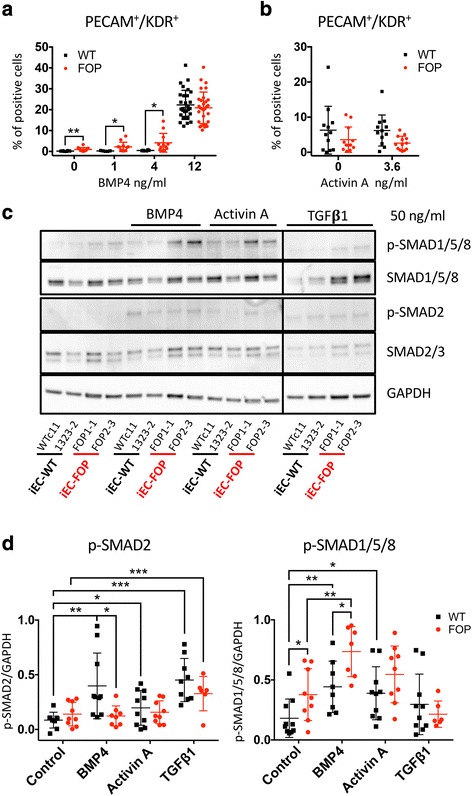
Fibrodysplasia ossificans progressiva (FOP) human induced pluripotent stem cell (hiPSC)-derived endothelial cells (iECs) form in conditions not permissive for controls and respond differently upon stimulation. **a** Wild type (WT) and FOP hiPSCs were differentiated as illustrated in Fig. 1d using different concentration of BMP4 (0, 1, 4, and 12 ng/ml). Shown is the mean percentage of PECAM^+^/KDR^+^ cells analyzed by fluorescent-activated cell sorting (FACS) at day 6 from three WT and four FOP hiPSC lines in at least three separate experiments for each group. Mean values were statistically different for 0, 1, and 4 ng/ml of BMP4. **b** WT and FOP hiPSCs were differentiated with or without supplemental Activin A. FACS analysis of PECAM^+^/KDR^+^ cells at day 6 did not show significant differences. **c** Representative western blot showing activation of SMAD pathways upon BMP4, Activin A, or TGFβ1 stimulation in WT and FOP iECs. **d** Western blot quantification of p-SMAD2 and p-SMAD1/5/8. Phosphorylation of SMAD2 was significantly increased in WT and FOP iECs treated with TGFβ1 compared to untreated cells. p-SMAD2 and p-SMAD1/5/8 protein expression were normalized to GAPDH protein expression. At least three separate experiments were run for WT and FOP iECs for each group. Error bars represent the mean ± one standard deviation. Mean values were statistically different by Student’s *t* test: * *p* < 0.05, ** *p* < 0.01, *** *p<0.005*

Because the ACVR1 R206H mutation is thought to up-regulate BMP pathway activity [14, 22, 23], we asked if FOP hiPSCs could form iECs in conditions not normally permissive for control cells. hiPSCs were cultured in varying concentrations of BMP4 during the initial EB formation (Fig. 1a). BMP4 removal significantly inhibited iEC formation from control hiPSCs, but the FOP hiPSCs still formed endothelial-like cells in those conditions (Fig. 4a). It is important to note that our differentiation protocol also contained Activin A. Thus, the formation of ECs at low BMP4 concentrations could result from increased sensitivity to BMP4 but also from aberrant activation of the SMAD1/5/8 pathway by Activin A in FOP cells [11, 12]. To test this possibility, we differentiated our WT and FOP hiPSCs in the absence of Activin A. We found no significant differences in the ability of WT or FOP hiPSCs to form iECs in the absence of or with the full dose (3.6 ng/ml) of Activin A (Fig. 4b). Together, these results suggest that FOP hiPSCs can form ECs even in conditions that are not normally permissive for WT cells and that the difference is likely due to BMP signaling rather than Activin A signaling.

### FOP iECs show increased SMAD1/5/8 signaling

To test if the increased plasticity in FOP ECs was due to activation of the BMP pathway, we next examined if SMAD signaling was activated in FOP iECs. When we initially examined SMAD signaling in FOP iECs cultured in fully supplemented media, we found no significant differences in SMAD1/5/8 activation when WT or FOP iECs were stimulated with BMP4 (Additional file 7: Figure S5A). However, after serum starvation, FOP iECs showed significantly increased phosphorylation of SMAD1/5/8 compared to WT iECs. The addition of BMP4 significantly increased the phosphorylation of SMAD2 and SMAD1/5/8 in WT iECs but only SMAD1/5/8 in FOP iECs (Fig. 4c, d). These results are consistent with the fact that BMPs have been reported to induce expression of EndMT markers in ECs [24] and that several other studies have reported increased SMAD2 signaling after BMP exposure in some cancer or transformed cells [25, 26].

**Fig. 5:**
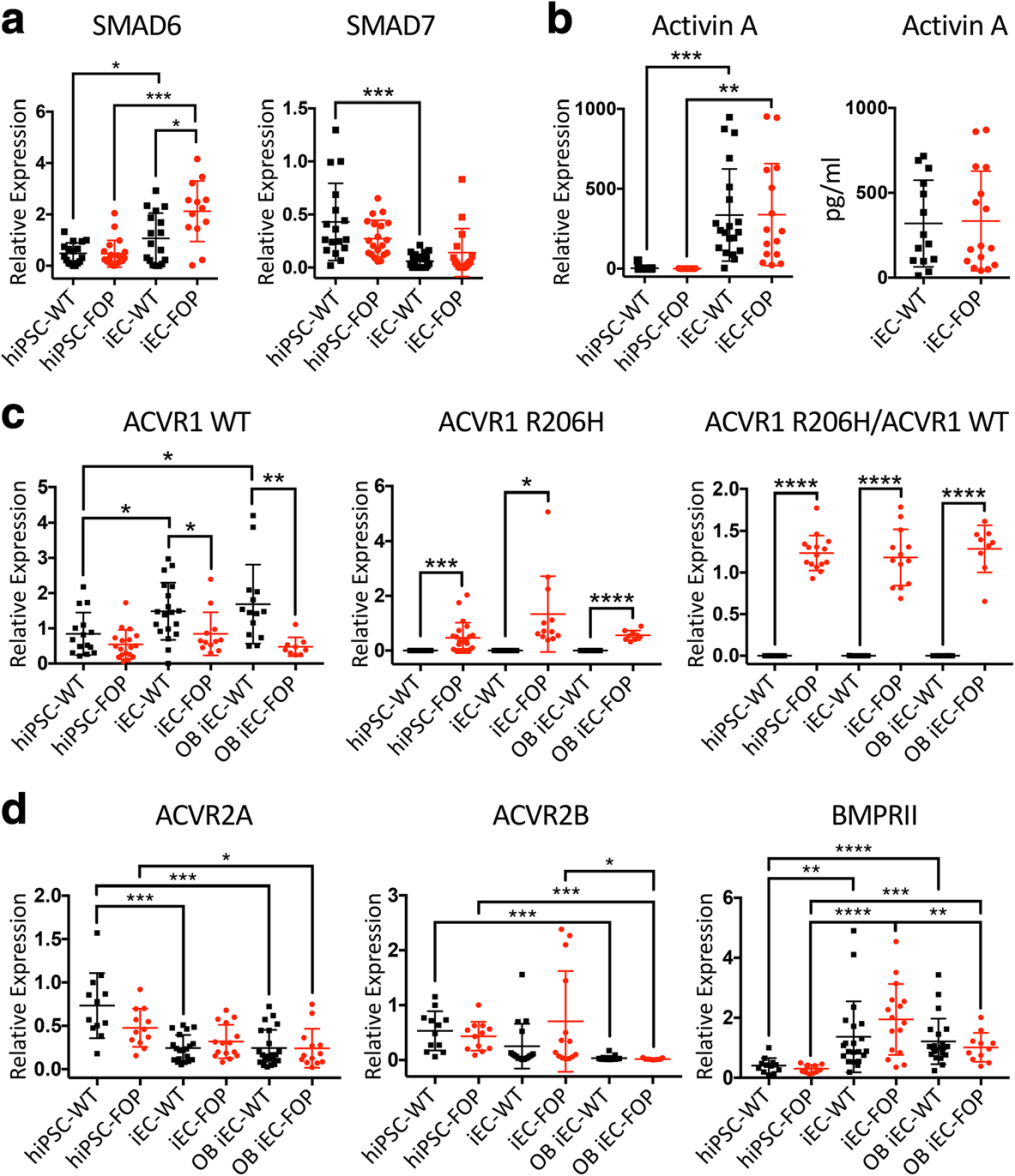
Cell type-specific expression of *SMAD*s and type I and II receptors. **a** Expression of the inhibitory *SMAD6* and *SMAD7* was higher in human induced pluripotent stem cell (hiPSC)-derived endothelial cells (iECs) than in hiPSCs. Also, *SMAD6* had significantly higher expression in fibrodysplasia ossificans progressiva (FOP) iECs. **b** Activin A gene expression levels were higher in iECs than in hiPSCs; however, there was no difference in Activin A levels between FOP and wild type (WT) by quantitative PCR or ELISA. **c** Gene expression analysis of WT *ACVR1*, *ACVR1* R206H, and the ratio of R206H/WT *ACVR1*. WT *ACVR1* expression was higher in iECs compared to hiPSCs. WT *ACVR1* levels were lower in FOP cells and *ACVR1* R206H was detected only in FOP cells, as expected. **d** *ACVR2A*, *ACVR2B*, and *BMPRII* gene expression analysis of WT and FOP hiPSCs, iECs, and iECs cultured in osteogenic media (OB iEC). Type II receptor expression was differentially expressed based on cell type but not on *ACVR1* R206H status. WT and FOP hiPSCs, iECs, and iECs cultured in osteogenic media were run in at least three separate experiments for each group. Error bars represent the mean ± one standard deviation. Mean values were statistically different by Student’s *t* test: * *p* < 0.05, ** *p* < 0.01, *** *p* < 0.005, **** *p* < 0.0001

Expression of *SMAD6*, which inhibits the phosphorylation of SMAD1/5/8 [27], and *SMAD7*, an inhibitor of SMAD2 and SMAD1/5/8 phosphorylation [27], was not significantly different between WT and FOP hiPSCs. Interestingly, *SMAD6* expression was significantly increased in FOP iECs compared to WT iECs. We also noticed that expression levels depended on cell type. Expression of *SMAD7* [27] was significantly lower in iECs compared to hiPSCs in WT cells but not in FOP cells (Fig. 5a). Conversely, expression of *SMAD6* [27] was significantly increased in iECs compared to hiPSCs. These findings suggest differential regulation of SMAD pathways dependent on cell type. In addition, SMAD2 and SMAD6 may have an unexpected autoregulatory effect on the SMAD1/5/8 pathway in FOP iECs.

Because recent studies [11, 12] showed that the ACVR1 R206H mutation may alter Activin A signaling in MSCs, we investigated if FOP iECs might produce or respond to Activin A. Although the expression of Activin A was significantly increased in iEC lines compared to hiPSC lines, we found no significant difference in Activin A mRNA or protein levels in culture media between WT and FOP iECs (Fig. 5b). FOP hiPSCs treated with Activin A showed increased SMAD1/5/8 phosphorylation compared to WT hiPSCs (Additional file 7: Figure S5B). In contrast, Activin A increased SMAD2 and SMAD1/5/8 phosphorylation in both WT and FOP iECs (Fig. 4c, d). FOP iECs treated with Activin A showed a non-significant trend towards increased SMAD1/5/8 phosphorylation as compared to WT iECs. We found no significant differences in SMAD2 phosphorylation between WT and FOP iECs treated with Activin A. This suggests that the BMP and Activin A ligands may differentially activate separate SMADs in FOP cells as compared to WT cells, thus leading to increased propensity for EndMT. To further elucidate if the up-regulation of mesenchymal genes found in FOP iECs could be caused by Activin A in the media, we treated our iEC cultures with the Activin A inhibitor follistatin. We found no significant differences between WT and FOP iECs treated with follistatin (Additional file 8: Figure S6). Thus, the up-regulation of mesenchymal gene expression in FOP iECs is not likely to be caused by Activin A.

TGFβ1 is known to play a major role in EC EndMT [28, 29]. Stimulating our iECs with TGFβ1 significantly increased SMAD2 phosphorylation in both WT and FOP iEC but did not change SMAD1/5/8 phosphorylation (Fig. 4d). This result suggests that FOP iECs respond in the same manner as WT iECs to TGFβ1 ligand.

### ACVR1 and type II receptors are differentially expressed in hiPSCs and iECs

Our unexpected findings of mild activation of SMAD1/5/8 by Activin A and SMAD2 by BMP4 (Fig. 4c, d) raised the question of whether cell type-specific expression of the ACVR1 receptors might be occurring. Expression of WT *ACVR1* was significantly increased in WT iECs and OB iECs compared to FOP iECs and FOP OB iECs (Fig. 5c). We also found a significant increase in WT *ACVR1* in WT iECs and in OB iECs compared to WT hiPSCs. However, expression of R206H *ACVR1* only showed a slight increase in iECs and the ratio of R206H to WT *ACVR1* showed no significant differences between hiPSC, iECs, and OB iECs (Fig. 5c).

Finally, we investigated if the differences seen in SMAD activation among different cell types might be related to altered expression of type II receptors known to bind to ACVR1, including ACVR2A, ACVR2B, and BMPRII [30]. Although WT and FOP cells expressed similar levels of *ACVR2A*, *ACVR2B*, and *BMPRII* (Fig. 5d), we again noted significant differences in the absolute expression levels between cells types. *ACVR2A* gene expression was significantly lower in WT iECs and OB iECs than in hiPSCs. *ACVR2B* was significantly lower only in OB iECs compared to WT and FOP hiPSCs and iECs. Interestingly, *BMPRII* expression levels were significantly lower in both WT and FOP hiPSCs in comparison to the other two cell types. Thus these differences in type II receptor expression between cell types, independent of the presence of the ACVR1 R206H allele, may account for the differences found in SMAD signaling in ECs compared to hiPSCs.

## Discussion

Aberrant BMP signaling can be found in many conditions of heterotopic ossification, including vascular calcification [31], atherosclerosis [32], and heterotopic ossification [33]. However, further delineation of the mechanisms inducing the abnormal bone formation has been limited by the broad expression patterns of the BMP ligands, potential differences in cell function between rodent models and humans, and the diversity of triggers that can incite heterotopic ossification. Genetic models using hiPSCs allows us to create human cell lineages with endogenous levels of gene expression for detailed studies.

**Fig. 6:**
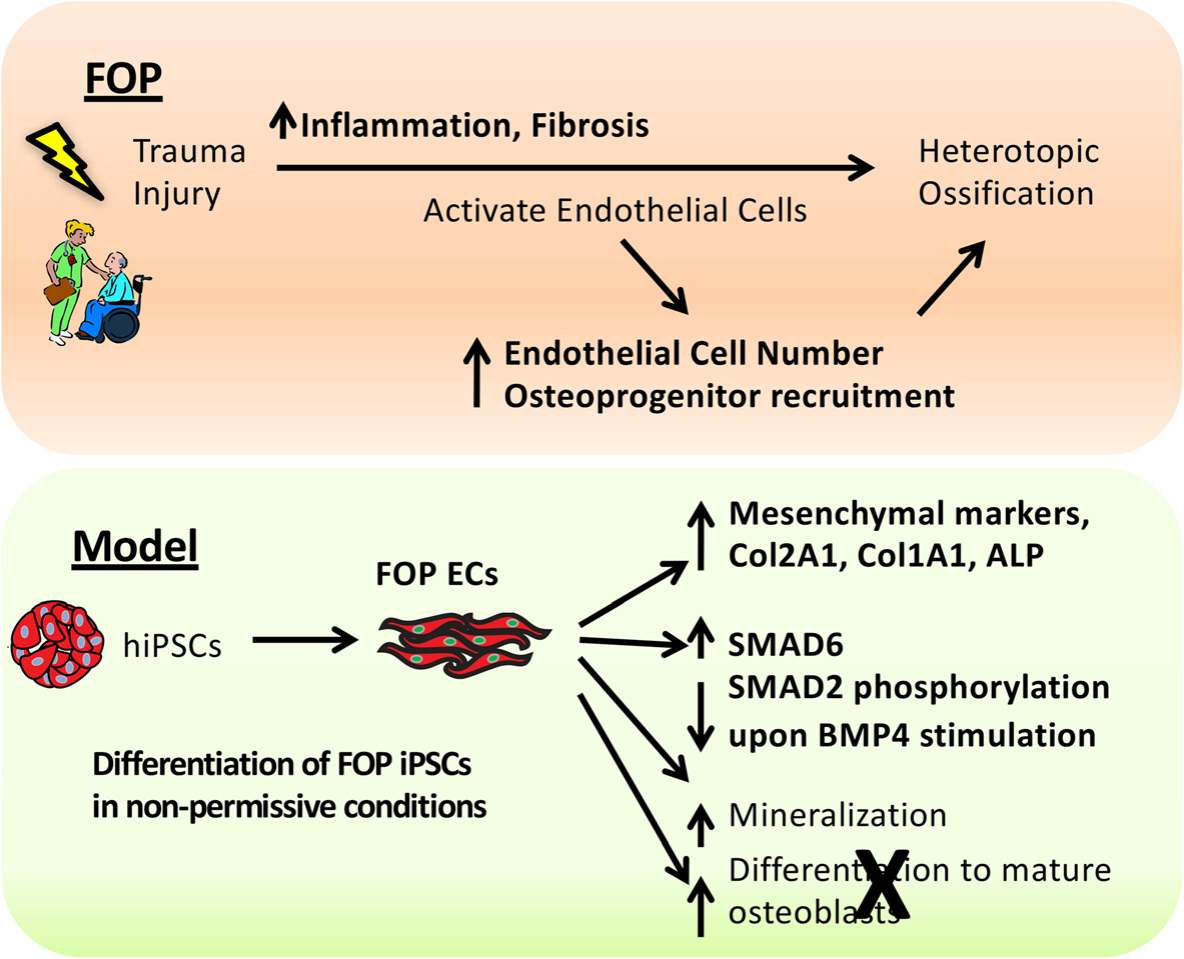
Summary of using human induced pluripotent stem cell (hiPSCs) to understand fibrodysplasia ossificans progressiva (FOP) R206H ACVR1 effects on human endothelial cell (EC) lineages. FOP hiPSCs are able to differentiate into ECs in a non-permissive condition and show increased osteogenic potential [14], which may reflect the increased number of ECs found in FOP lesions. FOP hiPSC-derived ECs (iECs) show increased expression of mesenchymal, extracellular matrix, chondrogenic, and osteogenic markers, which may play a major role in the tissue fibrosis found in patients with FOP during early stages of heterotopic ossification. FOP iECs show increased *SMAD6* gene expression and respond differently to different ligands such as BMP, which could be proposed as a mechanism for their loss of cellular commitment and their proficiency in undergoing endothelial-to-mesenchymal transition

Here, we used iECs to determine how ECs may contribute to heterotopic bone formation as a result of ACVR1 R206H expression. We show that iECs derived from FOP hiPSCs, which express physiological levels of the R206H *ACVR1* activating mutation, are useful tools for dissecting the cellular and molecular mechanisms that cause this debilitating disease. In addition, our study shows that the ACVR1 receptor may play a key role in regulating EC commitment, and that the ACVR1 R206H mutation may underlie the pathogenesis of abnormal bone formation by changing the propensity of ECs to undergo EndMT, possibly through variations in the cell’s responses to different ligands for ACVR1 (Fig. 6). We also show that ECs carrying the ACVR1 R206H receptor produce increased amounts of collagen proteins, likely contributing to the tissue fibrosis characteristic of heterotopic bone lesions.

Several studies suggest that multiple cell types, including ECs [9], mesenchymal progenitors [22], pericytes [20, 34], Tie2 cells [35, 36], and MSCs [37], may contribute to heterotopic ossification. Unlike a prior report [20], our detailed analysis revealed no evidence for impaired endothelial formation from FOP hiPSCs. In contrast, we found that FOP iECs could form in conditions not normally permissive for WT cells and that FOP iECs could express genes that may predispose them towards EndMT. Although our iEC model does not fully recapitulate the previously reported findings of increased mature osteogenesis by EC lineages in mice [9], our current in vitro conditions may not be optimized for creating mature mineral-depositing osteoblasts and thus limits our ability to understand how ACVR1 signaling affects the later steps of human osteogenesis. We also found an increase of ECs in FOP hiPSCs cultured in osteogenic media, suggesting that FOP ECs may not directly contribute to increased bone formation by transdifferentiation into osteoblasts but rather by acting as a supporting cell that helps establish a bone-forming niche for a bona fide osteogenic precursor. This possibility is supported by the significantly increased levels of collagen production found in FOP iECs. Furthermore, we were unable to create in vivo osteogenic implants made purely from the iECs (not shown), suggesting that a more complex cellular model may be needed. Addressing these important challenges is critical for future studies using in vitro directed differentiation approaches to study bone formation.

While our study cannot exclude roles for MSCs [22, 38] or pericytes [20] in FOP, PECAM^+^/KDR^+^ EC precursors could still be a potential cell source of osteoprogenitors. Our observed increase in some mesenchymal, chondrogenic, and osteogenic gene expression in FOP iECs suggests that *ACVR1* R206H can induce changes associated with cell fate. Cell lineage identity is necessary for homeostasis of most adult tissue and even subtle shifts in cell identity can severely impact tissues as they undergo regeneration after stress or injury. This loss of lineage determination, combined with the increased ability to make EC precursors in conditions not normally permissive in WT cells, may be critical early steps for developing heterotopic ossification in FOP. Indeed, a recent study has shown that increased cell plasticity can increase fibrogenesis in skeletal muscle, thus impairing normal tissue regeneration [39]. Our data that FOP iECs produce more extracellular matrix proteins provide supporting evidence that this may be happening in the early heterotopic ossification lesion, particularly given that fibrosis is a key early feature in FOP [40] and that changes in material properties can affect cell fate [41, 42]. We would speculate that the formation of fibrosis contributes to changes in cell fate [41, 42] and abnormal tissue regeneration in FOP *via* increased cell plasticity in skeletal muscle [39].

The recent identification of aberrant Activin A signaling by ACVR1 R206H [11, 38] using transgene expression models suggested that Activin A could be anti-angiogenic [43] and activated osteoblast differentiation and extracellular matrix mineralization [44]. Our results do not exclude FOP ECs as responders to Activin A, but indicate that FOP ECs do not contribute significantly to Activin A production. However, we found that FOP iECs responded differently from WT iECs when stimulated with BMP4 but not with Activin A or TGFβ1. We also saw an unexpected increase in SMAD1/5/8 signaling when WT hiPSCs were stimulated with Activin A. This is likely due to the different mRNA levels of *SMAD6* and *SMAD7* seen in hiPSCs versus ECs, as well as increased expression of *ACVR1* and *BMPRII* and decreased levels of *ACVR2A* and *ACVR2B* [45] in iECs versus hiPSCs. Surprisingly, we also found that BMP4 treatment of WT iECs increased SMAD2 phosphorylation. This could suggest that, despite BMP4’s low binding affinity to type II receptors [46], BMP4 may still be able to induce SMAD2 signaling in the absence of a high-affinity ligand such as Activin A. Indeed, a few studies have reported that BMPs could activate noncanonical SMAD2 [25, 26]. These findings suggest that individual cell types may respond differently to each ligand stimulus [47] and that specific clinical features of FOP may be caused by tissue-specific expression of either receptors or ligands.

## Conclusions

Together, our results show that EC lineages may contribute to heterotopic bone formation in FOP by priming an injury site with increased EC formation and changes in extracellular matrix production and fibrosis. In addition, BMPs and Activin A have a more complex interaction than previously thought, and differences in signaling via the type II receptors needs to be studied further in abnormal bone formation, tissue fibrosis, or the abnormal injury response in the pathogenesis of heterotopic bone formation. The broad expression of BMP and Activin A, particularly after injury, suggests that these mechanisms may be more generally applicable to non-genetic forms of heterotopic ossification [48] and potentially identify a new role for endothelial lineage cells in both normal and pathogenic osteogenesis.

## Abbreviations

ACVR1, Activin A type 1 receptor; ALPL, alkaline phosphatase; bFGF, Basic fibroblast growth factor; BMP, Bone morphogenetic protein; EB, embryoid bodies; EC, endothelial cell; ECM, endothelial cell medium; EndMT, endothelial-to-mesenchymal transition; FOP, fibrodysplasia ossificans progressiva; hESC, human embryonic stem cell; hiPSC, human induced pluripotent stem cell; HUVEC, human umbilical vein endothelial cells; ICAM-1, Intercellular adhesion molecule; iEC, hiPSC-derived endothelial cell; KDR, Kinase insert domain receptor; MSC, mesenchymal stem cell; MTG, PBS, phosphate-buffered saline; PECAM, Platelet endothelial cell adhesion molecule; TGFβ1, Transforming growth factor β1; TNFα, Tumor necrosis factor α; VE-cadherin, Vascular endothelial cadherin; VEGF, Vascular endothelial cell growth factor; WT, wild type

## Additional files

Additional file 1: Table S1. Taqman Primers-gene expression. (DOCX 37 kb) Additional file 2: Table S2. Sybr Green primers-gene expression. (DOC 27 kb) Additional file 3: Figure S1. Increased endothelial marker expression during osteoblast differentiation of hiPSCs and differentiation of hiPSCs into ECs. (A) Quantitation of *PECAM* and *VE-Cadherin* gene expression by qPCR during 12 days of culture in osteogenic medium. (B) FACS analysis of mean percentage of cells expressing both PECAM and KDR on day 6 and 12 of osteogenic differentiation of three WT and three FOP hiPSCs lines with at least three replicates for each cell line. Error bars represent mean ± one SD. * *p* < 0.05, ** *p* < 0.01 by Student’s *t* test. (C) Mean percentage of cells co-expressing MSC markers (CD90^+^/CD73^+^/CD105^+^) on day 12 of osteogenic differentiation of three WT and three FOP hiPSCs lines. Error bars represent mean ± one standard deviation of at least three independent replicates for each of the three WT and four FOP cell lines. MSCs were not significantly increased in FOP hiPSCs during osteogenic differentiation by Student’s *t* test. (TIF 116 kb) Additional file 4: Figure S2. Functional characterization of WT and FOP iECs. (A) Both WT and FOP iECs have the ability to form tube-like structures in an in vitro Matrigel assay. Scale bars, 300 μm. (B) WT and FOP iECs showed activation upon TNFα stimulation. WT and FOP iECs were stimulated with 10 ng/ml TNFα overnight. Cells co-expressing ICAM-1 and PECAM were quantified by FACS analysis. ** *p* < 0.01, *** *p* < 0.005, **** *p* < 0.0001 by Student’s *t* test. Error bars represent mean ± one standard deviation of at least three independent replicates for each HUVEC WT and FOP iEC line. (TIF 301 kb) Additional file 5: Figure S3. WT and FOP iECs show similar qualitative migration properties to VEGF in a transwell assay. Scale bars, 100 μm. (TIF 1177 kb) Additional file 6: Figure S4. Characterization of osteogenic and chondrogenic potential of FOP iECs. Gene expression analysis of *SMA*, *SOX9*, *MMP10*, *SP7*, and *RUNX2* by qPCR of WT and FOP iECs. None of these gene expression levels were statistically different by Student’s *t* test. Error bars represent mean ± one standard deviation of at least three independent replicates for each of the three WT and four FOP iEC lines. (TIF 158 kb) Additional file 7: Figure S5. SMAD signaling activity of hiPSCs and iECs. (A) Quantitation of SMAD1/5/8 phosphorylation without serum starvation upon BMP4 stimulation in WT and FOP iECs. p-SMAD1/5/8 protein expression was normalized to GAPDH protein expression. At least three separate experiments were run for WT and FOP iECs for each group. Error bars represent mean ± one standard deviation. Mean values were not statistically significant by Student’s *t* test. (B) Representative western blot showing activation of SMAD2 and SMAD1/5/8 pathways upon BMP4 and Activin A stimulation in WT and FOP hiPSCs. Samples were derived from the same experiment and processed in parallel. (TIF 221 kb) Additional file 8: Figure S6. Inhibition of Activin A by follistatin does not down-regulate mesenchymal genes in FOP iECs. iECs were treated with follistatin. Error bars represent mean ± one standard deviation of at least two independent replicates for two WT and three FOP iEC lines; * *p* < 0.05, ** *p* < 0.01 by Student’s *t* test. (TIF 99 kb)
