# Supplementary material for: The ACVR1 R206H mutation found in fibrodysplasia ossificans progressiva increases human induced pluripotent stem cell-derived endothelial cell formation and collagen production through BMP-mediated SMAD1/5/8 signaling
Source: Stem Cell Res Ther. 2016 Aug 17;7:115. doi: 10.1186/s13287-016-0372-6 (PMC4988052; doi:10.1186/s13287-016-0372-6)
Supplement: Additional file 1: Table S1. — Taqman Primers-gene expression. (DOCX 37 kb) [file 13287_2016_372_MOESM1_ESM.docx]

**Table S1.** Taqman Primers-gene expression

| Sequence | Assay ID |
| --- | --- |
| ACVR2A | Hs00155658_m1 |
| ACVR2B | Hs00609603_m1 |
| Alkaline Phosphatase | Hs01029144_m1 |
| BMPR2 | Hs00176148_m1 |
| CD34 | Hs00990732_m1 |
| VE-Cadherin | Hs00901463_m1 |
| COL1A1 | Hs01076780_g1 |
| COL2A1 | Hs01060334_g1 |
| GAPDH | Hs02758991_g1 |
| INHBA (Activin A) | Hs1081598_m1 |
| PECAM-1 | Hs00169777_m1 |
| RUNX2 | Hs00231692_m1 |
| S100A4 (FSP-1) | Hs00243202_m1 |
| SMAD6 | Hs00178579_m1 |
| SMAD7 | Hs00998193_m1 |
| SOX9 | Hs00165814_m1 |
| SP7 | Hs01866874_s1 |
| SMA | Hs01038777_g1 |
| TIE-2 | Hs00945146_m1 |
| vWF | Hs00169795_m1 |
