# Supplementary material for: The ACVR1 R206H mutation found in fibrodysplasia ossificans progressiva increases human induced pluripotent stem cell-derived endothelial cell formation and collagen production through BMP-mediated SMAD1/5/8 signaling
Source: Stem Cell Res Ther. 2016 Aug 17;7:115. doi: 10.1186/s13287-016-0372-6 (PMC4988052; doi:10.1186/s13287-016-0372-6)
Supplement: Additional file 2: Table S2. — Sybr Green primers-gene expression. (DOC 27 kb) [file 13287_2016_372_MOESM2_ESM.docx]

**Table S2.** Sybr Green primers-gene expression

| Sybr Green Pprimers | Forward Primer | Reverse Primer |
| --- | --- | --- |
| hACVR1 WT | TGGTACAAAGAACAGTGGCTAG | CCATACCTGCCTTTCCCGA |
| hACVR1 R206H | TGGTACAAAGAACAGTGGCTTA | CCATACCTGCCTTTCCCGA |
| hGAPDH | AGATCATCAGCAATGCCTCCTG | ATGGCATGGACTGTGGTCATG |

References: J Kaplan et al, Gene Ther, 2011; Liori, et al., JBMR, 2006.
