# Supplementary figures and images for: The ACVR1 R206H mutation found in fibrodysplasia ossificans progressiva increases human induced pluripotent stem cell-derived endothelial cell formation and collagen production through BMP-mediated SMAD1/5/8 signaling
Source: Stem Cell Res Ther. 2016 Aug 17;7:115. doi: 10.1186/s13287-016-0372-6 (PMC4988052; doi:10.1186/s13287-016-0372-6)

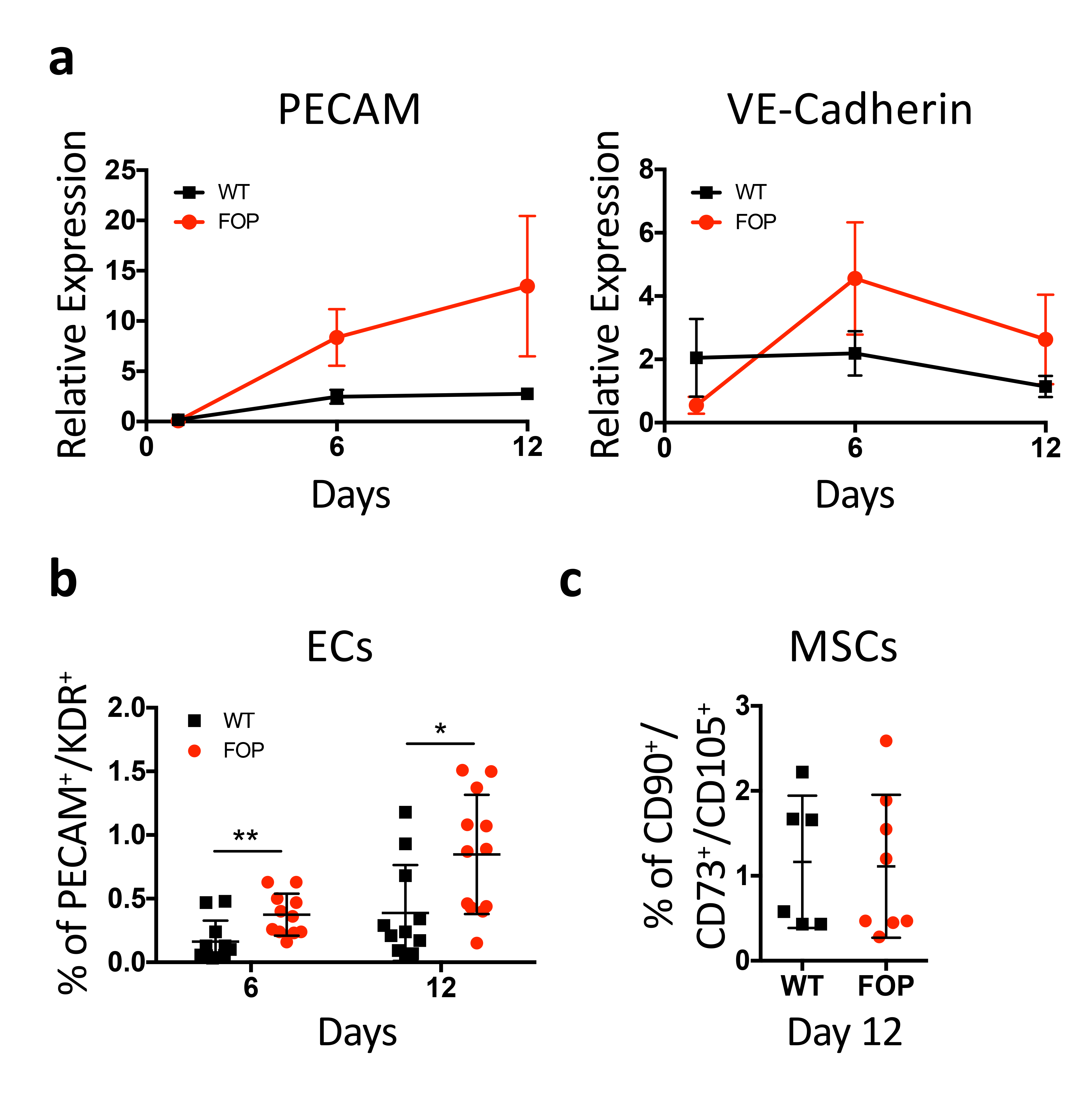

Supplement: Additional file 3: Figure S1. — Increased endothelial marker expression during osteoblast differentiation of hiPSCs and differentiation of hiPSCs into ECs. (A) Quantitation of PECAM and VE-Cadherin gene expression by qPCR during 12 days of culture in osteogenic medium. (B) FACS analysis of mean percentage of cells expressing both PECAM and KDR on day 6 and 12 of osteogenic differentiation of three WT and three FOP hiPSCs lines with at least three replicates for each cell line. Error bars represent mean ± one SD. * p < 0.05, ** p < 0.01 by Student’s t test. (C) Mean percentage of cells co-expressing MSC markers (CD90+/CD73+/CD105+) on day 12 of osteogenic differentiation of three WT and three FOP hiPSCs lines. Error bars represent mean ± one standard deviation of at least three independent replicates for each of the three WT and four FOP cell lines. MSCs were not significantly increased in FOP hiPSCs during osteogenic differentiation by Student’s t test. (TIF 116 kb) [file 13287_2016_372_MOESM3_ESM.tif]

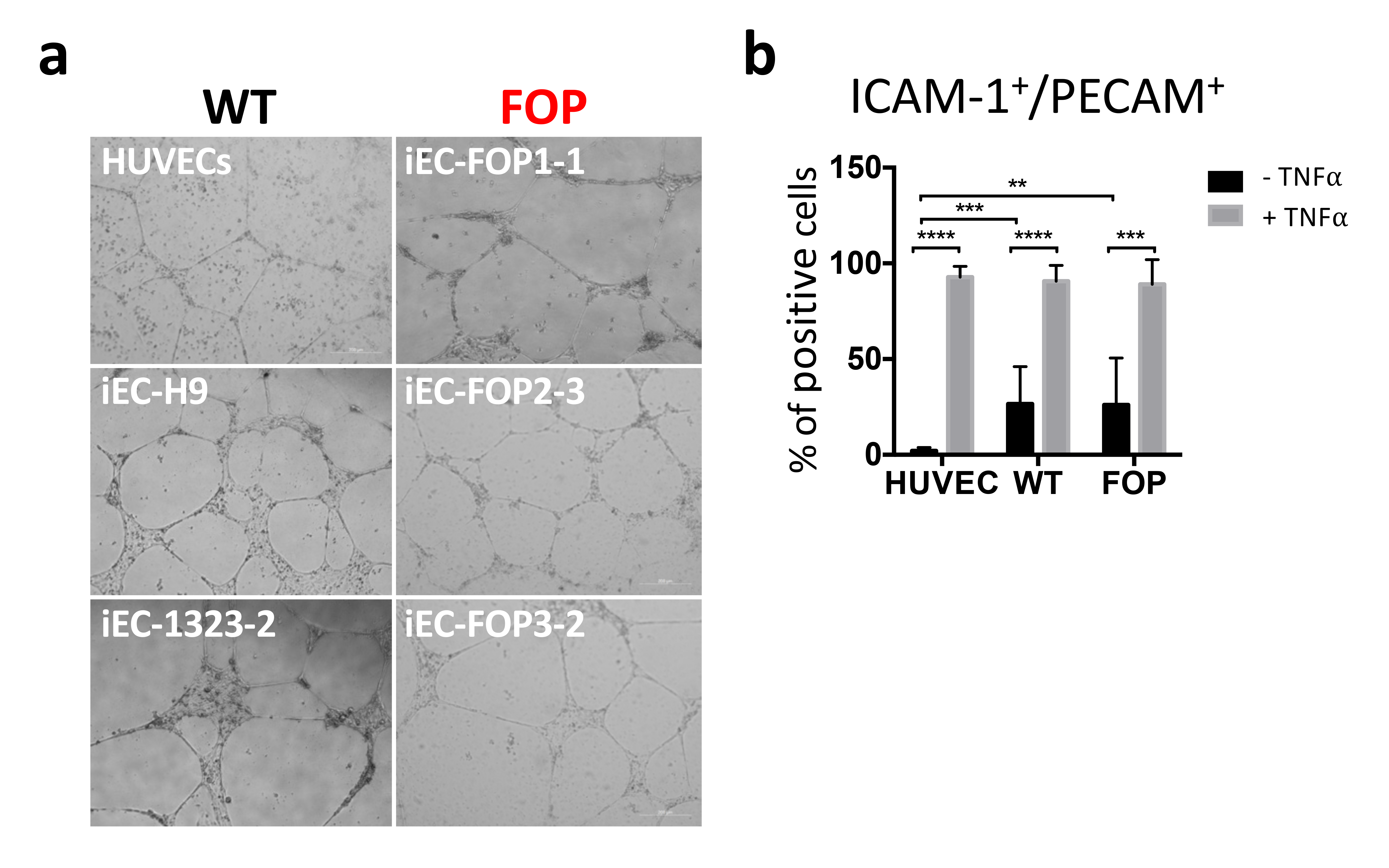

Supplement: Additional file 4: Figure S2. — Functional characterization of WT and FOP iECs. (A) Both WT and FOP iECs have the ability to form tube-like structures in an in vitro Matrigel assay. Scale bars, 300 μm. (B) WT and FOP iECs showed activation upon TNFα stimulation. WT and FOP iECs were stimulated with 10 ng/ml TNFα overnight. Cells co-expressing ICAM-1 and PECAM were quantified by FACS analysis. ** p < 0.01, *** p < 0.005, **** p < 0.0001 by Student’s t test. Error bars represent mean ± one standard deviation of at least three independent replicates for each HUVEC WT and FOP iEC line. (TIF 301 kb) [file 13287_2016_372_MOESM4_ESM.tif]

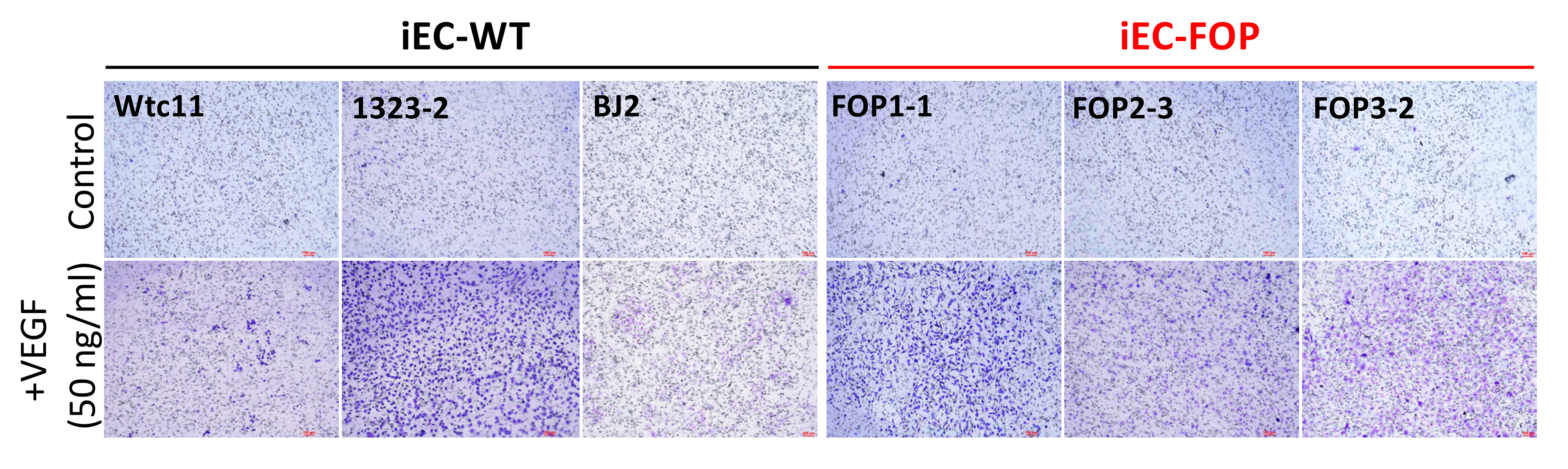

Supplement: Additional file 5: Figure S3. — WT and FOP iECs show similar qualitative migration properties to VEGF in a transwell assay. Scale bars, 100 μm. (TIF 1177 kb) [file 13287_2016_372_MOESM5_ESM.tif]

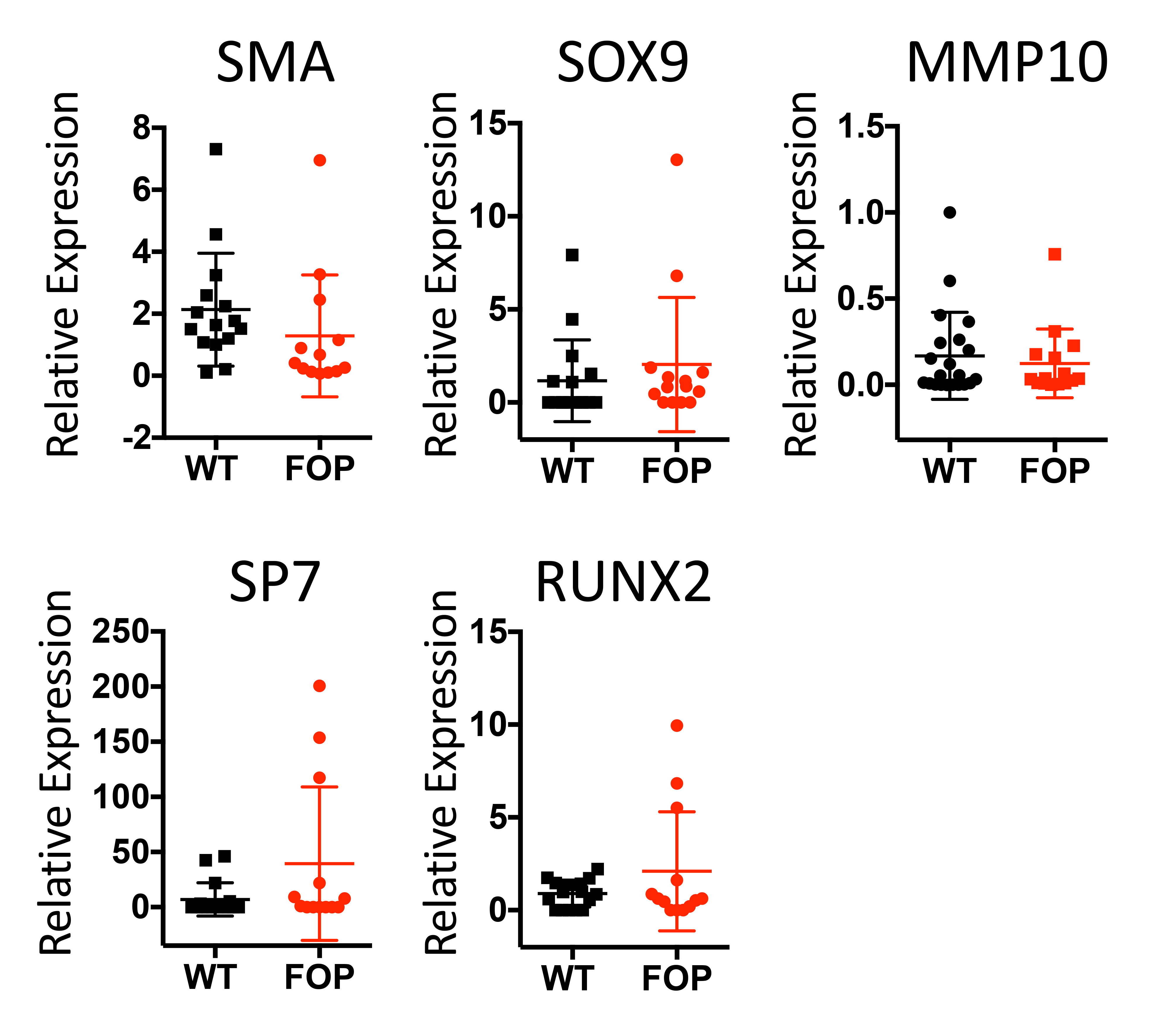

Supplement: Additional file 6: Figure S4. — Characterization of osteogenic and chondrogenic potential of FOP iECs. Gene expression analysis of SMA, SOX9, MMP10, SP7, and RUNX2 by qPCR of WT and FOP iECs. None of these gene expression levels were statistically different by Student’s t test. Error bars represent mean ± one standard deviation of at least three independent replicates for each of the three WT and four FOP iEC lines. (TIF 158 kb) [file 13287_2016_372_MOESM6_ESM.tif]

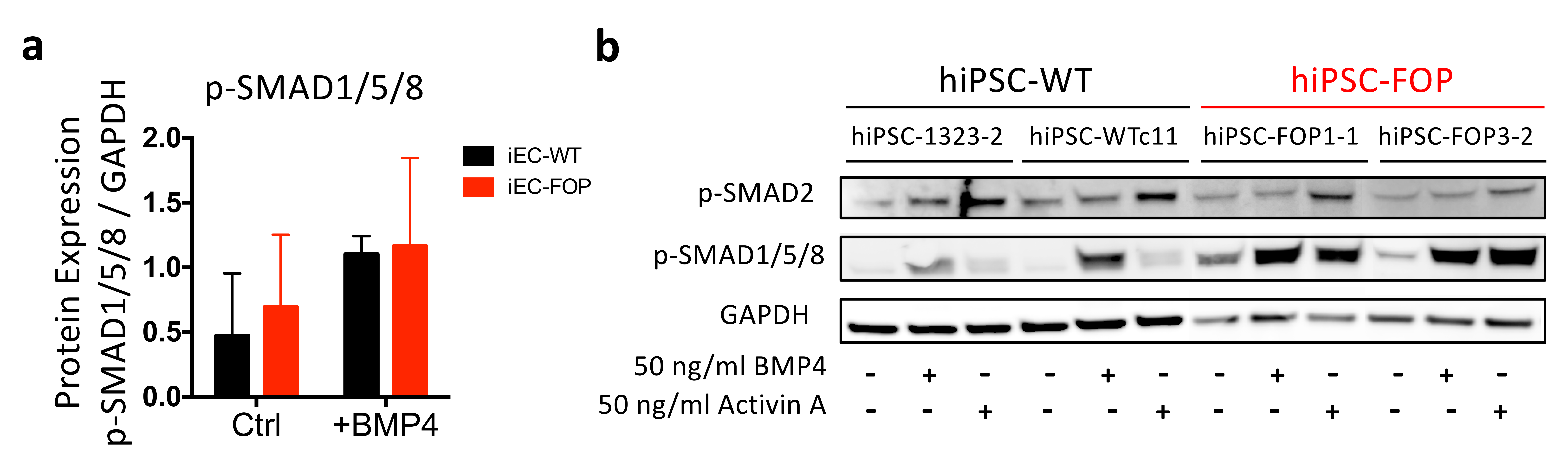

Supplement: Additional file 7: Figure S5. — SMAD signaling activity of hiPSCs and iECs. (A) Quantitation of SMAD1/5/8 phosphorylation without serum starvation upon BMP4 stimulation in WT and FOP iECs. p-SMAD1/5/8 protein expression was normalized to GAPDH protein expression. At least three separate experiments were run for WT and FOP iECs for each group. Error bars represent mean ± one standard deviation. Mean values were not statistically significant by Student’s t test. (B) Representative western blot showing activation of SMAD2 and SMAD1/5/8 pathways upon BMP4 and Activin A stimulation in WT and FOP hiPSCs. Samples were derived from the same experiment and processed in parallel. (TIF 221 kb) [file 13287_2016_372_MOESM7_ESM.tif]

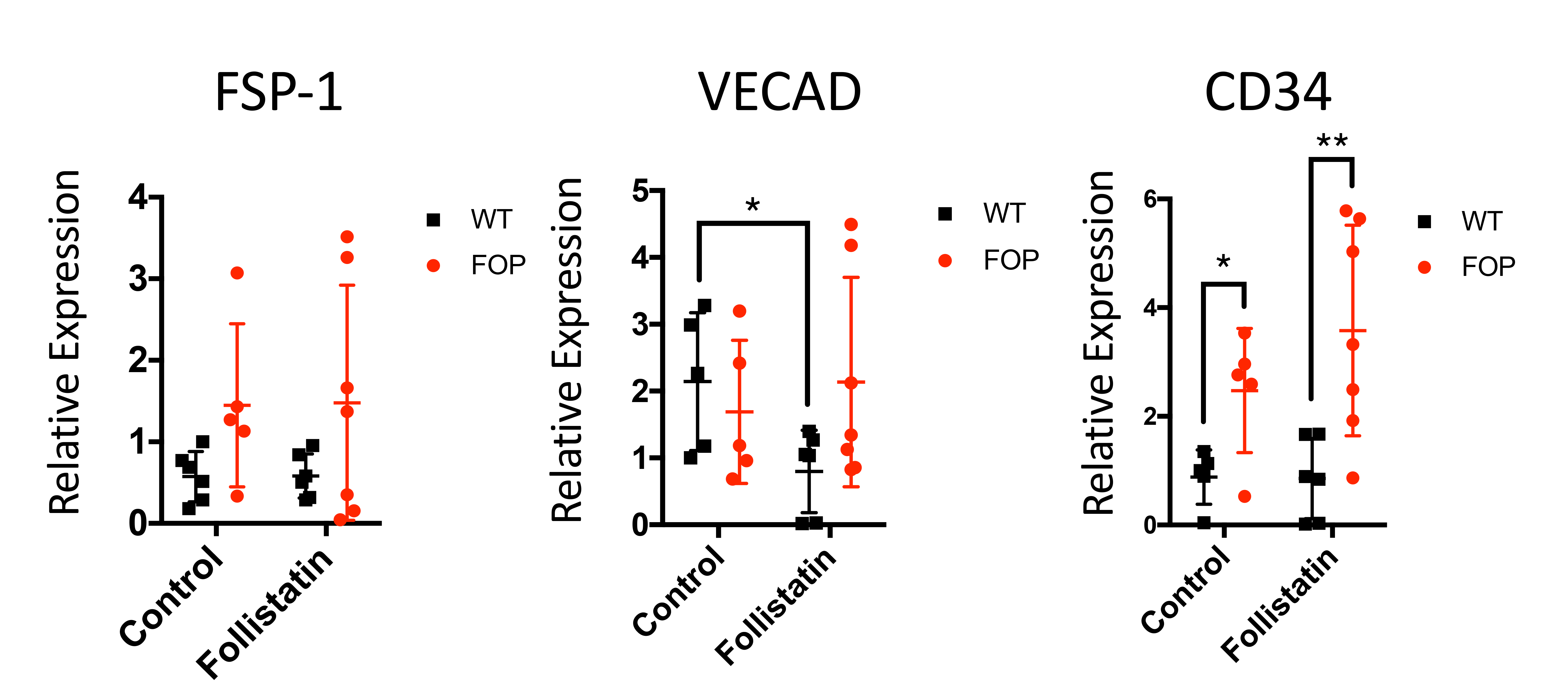

Supplement: Additional file 8: Figure S6. — Inhibition of Activin A by follistatin does not down-regulate mesenchymal genes in FOP iECs. iECs were treated with follistatin. Error bars represent mean ± one standard deviation of at least two independent replicates for two WT and three FOP iEC lines; * p < 0.05, ** p < 0.01 by Student’s t test. (TIF 99 kb) [file 13287_2016_372_MOESM8_ESM.tif]
